# Fault Diagnosis Method for Reciprocating Compressors Based on Spatio-Temporal Feature Fusion

**DOI:** 10.3390/s26030798

**Published:** 2026-01-25

**Authors:** Haibo Xu, Xiaolong Ji, Xiaogang Qin, Weizheng An, Fengli Zhang, Lixiang Duan, Jinjiang Wang

**Affiliations:** 1 Department of Safety Engineering, College of Mechanical and Transportation Engineering, China University of Petroleum, Beijing 102249, China2024210716@student.cup.edu.cn (X.J.);; 2 CNOOC China Limited Beijing Research Center, Beijing 100028, China

**Keywords:** reciprocating compressor, spatio-temporal features, feature extraction, feature fusion, fault diagnosis

## Abstract

Reciprocating compressors, which serve as core equipment in the petrochemical and natural gas transmission sectors, operate under prolonged variable loads and high-frequency impact conditions. Critical components, such as valves and piston rings, are prone to failure. Existing fault diagnosis methods suffer from inadequate spatio-temporal feature extraction and neglect spatio-temporal correlations. To address this, this paper proposes a spatio-temporal feature fusion-based fault diagnosis method for reciprocating compressors. This method constructs a spatio-temporal feature fusion model (STFFM) comprising three principal modules: First, a spatio-temporal feature extraction module employing a multi-layered stacked bidirectional gated recurrent unit (BiGRU) with batch normalisation to uncover temporal dependencies in long-term sequence data. A graph structure is constructed via k-nearest neighbours (KNN), and an enhanced graph isomorphism network (GIN) is integrated to capture spatial domain fault information variations. Second, the spatio-temporal bidirectional attention-gated fusion module employs a bidirectional multi-head attention mechanism to enhance temporal and spatial features. It incorporates a cross-modal gated update mechanism and learnable weight parameters to dynamically retain the highly discriminative features. Third, the classification output module enhances the model’s generalisation capability through multi-layer fully connected layers and regularisation design. Research findings demonstrate that this approach effectively integrates spatio-temporal coupled fault features, achieving an average accuracy of 99.14% on an experimental dataset. This provides an effective technical pathway for the precise identification of faults in the critical components of reciprocating compressors.

## 1. Introduction

Reciprocating compressors, which serve as core equipment in critical industrial sectors such as petrochemicals and natural gas transmission, endure harsh operating conditions characterised by variable loads and high-frequency impacts owing to their reciprocating motion structure. This renders key components prone to failure, necessitating highly efficient and precise fault diagnosis technology [1].

Traditional diagnostic methods for reciprocating compressor faults primarily rely on two technical approaches: firstly, signal processing-based diagnostics. This involves capturing single physical signals, such as vibration, pressure, or temperature, and then employing techniques such as Fourier transforms, wavelet analysis, or empirical mode decomposition to extract time-domain or frequency-domain features. These features are subsequently analysed using classifiers, such as support vector machines (SVMs) or artificial neural networks (ANNs), to identify faults. Li et al. [2] employed the empirical mode decomposition algorithm to decompose compressor unit fault vibration signals into a series of eigenmode functions. Subsequently, a backpropagation neural network was used to classify and identify fault characteristics, thereby achieving fault diagnosis for reciprocating compressor units. Qi et al. [3] proposed a fault diagnosis method for reciprocating compressors, which involved first denoising the raw data and training a sparse coding dictionary, followed by the use of an SVM to identify and classify potential faults. Wang et al. [4] employed variational mode decomposition coupled with SDP transformation to generate fault fusion images, subsequently utilising CNN to recognise the Variational Mode Decomposition-Symmetrised Dot Pattern (VMD-SDP) fusion images for diagnosing reciprocating compressors.

Such methods have played a certain role in early diagnosis, but there is room for improvement. On the one hand, the failure evolution process of reciprocating compressors exhibits pronounced temporal correlation and spatial distribution characteristics. For instance, piston ring wear progressively intensifies with operating time, whereas exhaust pressure fluctuations and valve plate impact sounds demonstrate distinct spatial domain distributions. Relying solely on single-dimensional and positional signal features makes it challenging to comprehensively characterise the multi-source coupling properties of failures, often resulting in either missing or redundant feature information. Secondly, mechanism-based diagnostic methods establish thermodynamic and kinetic models of reciprocating compressors to analyse parameter deviations during fault conditions, thereby pinpointing the fault location. Zhang et al. [5] calculated and validated flow coefficients using a three-dimensional model and analysed variations in thermodynamic parameters under abnormal operating conditions, such as inconsistent suction valve lift. This provides guidance for compressor design and fault diagnosis. Wang et al. [6] proposed a valve fault diagnosis method for reciprocating compressors that integrated acoustic emission signals with simulated valve motion. By analysing the acoustic emission signals within the crankshaft angular domain, the method captures the actual valve operating conditions. Simulated valve motion predicts the operational state and provides reference values for normal valve opening and closing positions to aid in diagnosis. Li et al. [7] proposed a non-destructive fault diagnosis method for reciprocating compressors based on piston rod strain to obtain p-V diagrams. This approach constructs a p-V diagram reconstruction algorithm by identifying key characteristic points that reflect valve opening and closing events on the piston rod load curve. Although these methods have clear physical significance, the internal flow field within reciprocating compressors is complex. Furthermore, during actual operation, factors such as the composition of the medium and ambient temperature introduce disturbances, making it difficult to guarantee model accuracy.

With the advancement of artificial intelligence technology, multisensor data fusion has opened new avenues for fault diagnosis in reciprocating compressors. On the one hand, deploying vibration, pressure, and temperature sensors at critical locations, such as the compressor cylinder and crankcase, enables synchronous multidimensional and multispatial data acquisition, providing comprehensive data support for capturing fault characteristics. In contrast, deep learning technology possesses robust capabilities for automatic feature extraction and nonlinear mapping, enabling the mining of implicit fault correlation information from vast datasets. Tian et al. [8] proposed a multi-segment attention long short-term memory network (MA-LSTMs) based on spatio-temporal multi-information fusion for the operational condition monitoring of reciprocating compressors on offshore oil platforms. Tang et al. [9] employed the Sparrow Search algorithm to optimise a kernel support vector machine for constructing a diagnostic model, efficiently integrating multi-source signals from compressors.

Moreover, existing diagnostic methods predominantly focus on sequence modelling in the time domain or feature concatenation in the spatial domain. Regarding temporal modelling, Cabrera et al. [10] iteratively trained LSTM models from vibration signal time-series while employing Bayesian optimisation to constrain the search space for hyperparameter optimisation, thereby capturing the dynamic temporal characteristics of faults. Zhao et al. [11] proposed an adaptive variational time-domain decomposition (VTDD) method for processing multi-impact vibration signals in reciprocating machinery, which can effectively identify fault-induced additional impacts and accurately extract time-domain impact features.

Regarding feature extraction in the spatial domain, Zhou et al. [12] proposed an adaptive stochastic resonance method based on GANs. The GAN generator generates SR parameters based on the time-domain and frequency-domain features of the vibration signal, whereas the discriminator optimises the model by concatenating the features of both real and generated SR parameters. This ultimately achieves the dynamic generation and concatenation of the spatial domain features. In recent years, spatial domain feature extraction methods that employ graph convolutional neural networks have also been applied. He et al. [13] proposed an adaptive graph small-frame convolutional network fault diagnosis framework that utilises small-frame transform techniques to reduce signal interference and enhance the spatial feature learning capability of the model. However, these approaches fail to adequately account for the spatio-temporal coupling of fault characteristics. For instance, a valve leakage fault not only increases the temporal peak amplitude of the vibration signals at the valve location but also induces spatial variations in the pressure signals between the cylinder and valve chamber. Moreover, these spatio-temporal features exhibit dynamic evolution patterns as the fault progresses. Neglecting the intrinsic correlation between spatio-temporal characteristics readily leads to increased redundancy and diminished discriminative power in fused features.

To address the inadequacies of traditional methods in spatio-temporal feature extraction and the neglect of spatio-temporal correlations in existing modelling approaches, this paper constructs a spatio-temporal feature fusion model (STFFM).

(1) The spatio-temporal feature extraction component employs a multi-layered stacked bidirectional gated recurrent unit (BiGRU) incorporating batch normalisation. to enhance the extraction of temporal dependencies from long-term sequence data. Concurrently, a graph structure is constructed using K-Nearest Neighbours (KNN) and combined with an improved graph isomorphism network (GIN) to transform one-dimensional signals into graph data, thereby capturing spatial variations in fault information. The robustness of the spatial features is further enhanced through a multilayer perceptron (MLP) layer with an enhanced and regularised design.
(2) The spatio-temporal fusion component implements a bidirectional multi-head attention mechanism to enhance temporal and spatial features, generating augmented features from dual perspectives. This is combined with a cross-modal gated update mechanism and learnable weight parameters to dynamically retain the highly discriminative features.
(3) The classification output section employs multilevel fully connected layers and a regularisation design. This approach prevents the loss of high-dimensional feature information while enhancing the model’s generalisation capability, ensuring precise fault classification.

This diagnostic methodology provides an efficient analytical and interpretative solution for the high-dimensional, multi-source, and heterogeneous data gathered by sensors, representing a practical extension of sensor applications in the field of equipment condition monitoring.

The remainder of this paper is organised as follows: Section 2 introduces the theoretical foundations of spatio-temporal feature extraction. Section 3 details the model construction methodology and fault diagnosis procedures. Section 4 analyses the diagnostic performance of the proposed method through case studies. Section 5 discusses the effectiveness and superiority of the proposed approach via ablation and comparative experiments. Section 6 summarises the conclusions of the study.

## 2. Theoretical Basis

### 2.1. Graph Neural Network

The graph isomorphism network GIN is a variant of the graph neural network GNN proposed by Xu et al. in 2018 [14], designed to address the graph isomorphism problem, which determines whether two structurally distinct graphs share identical topological structures. Its methodology is grounded in the classical WL graph isomorphism test algorithm. This algorithm iteratively aggregates a node’s intrinsic features with those of its neighbours, incorporating learnable aggregation weights. This enables the model to distinguish between different graph structures, thereby yielding highly discriminative spatial domain features.

The core concept of GIN is k-order neighbour aggregation: the feature of a node at layer *k* is obtained by aggregating its own feature at layer *k* − 1 with the features at layer *k* − 1 of all its neighbours. After *k* iterations, the node feature incorporates structural information from k-order neighbours, ultimately yielding the feature of the entire graph through global pooling. The GIN transforms the WL algorithm into a learnable neural network model using Equation (1):

(1) $$h_{v}^{\left(k\right)}={\mathrm{MLP}}^{\left(k\right)}\left(\left(1+\epsilon^{\left(k\right)}\right)\cdot h_{v}^{\left(k-1\right)}+\sum_{u\in N(v)}h_{u}^{\left(k-1\right)}\right)$$

where $h_{v}^{\left(k\right)}$ denotes the feature representation of node *v* at layer *k*; N(v) represents the neighbourhood set of node *v*; $\epsilon^{\left(k\right)}$ is the learnable parameter, used to adjust the weighting of its own features; ${\mathrm{MLP}}^{\left(k\right)}$ is a multilayer perceptron employing nonlinear transformations to enhance the model’s expressive capability.

In Equation (1), $\left(1+\epsilon \right)\cdot h_{v}+\sum h_{u}$ simulates the operation in the Weighted Layer-wise (WL) algorithm by concatenating its own labels with those of its neighbours, achieving feature aggregation and discrimination through linear transformations. The MLP, which serves as a nonlinear transformation, replaces the hash mapping in the WL algorithm. It has stronger fitting capabilities and can learn more complex feature mappings.

To obtain a representation of the entire graph rather than individual nodes, the GIN employs a readout function to aggregate the final features of all nodes:

(2) $$h_{G}=\mathrm{Readout}\left(\left\{h_{v}^{\left(K\right)}|v\in G\right\}\right)$$

Reading functions typically employ multi-set aggregation to calculate the sum, mean, or maximum value of all node features. By concatenating node features from different layers through multiple rounds of iteration, similar to the WL algorithm, they further enhanced the discriminative capability.

### 2.2. Bidirectional Gated Recurrent Unit

In the field of fault diagnosis, the core requirement is to capture characteristic changes in equipment transitioning from normal to faulty states through temporal monitoring data, such as vibration, temperature, current, and pressure, thereby enabling precise differentiation between various faults. The bidirectional gated recurrent unit BiGRU, owing to its efficient capability for capturing bidirectional temporal dependencies, has emerged as one of the core models for processing time-domain data [15]. BiGRU is a sequence model extended from the gated recurrent unit (GRU), with its core concept being the simultaneous utilisation of both forward and backward sequence information to address the unidirectional dependency issue inherent in traditional models [16].

For a forward time-series $X=x_{1},x_{2},\ldots ,x_{t}$, the forward computation of the BiGRU model can be expressed as follows:

(3) $${\vec{h}}_{t}=\mathrm{GRU}({\vec{h}}_{t-1},x_{t})$$

For a reverse time-series $X=x_{t},x_{t-1},\ldots ,x_{1}$, the backward computation of the BiGRU model can be expressed as follows:

(4) $${\overset{\leftarrow }{h}}_{t}=\mathrm{GRU}({\overset{\leftarrow }{h}}_{t+1},x_{t})$$

where ${\vec{h}}_{t}$ and ${\overset{\leftarrow }{h}}_{t}$ denotes the forward and backward hidden states, respectively; GRU represents the GRU unit; and *x_t_* denotes the *t*-th element in the input sequence. At each time step *t*, the forward hidden state ${\vec{h}}_{t}$ and backward hidden state ${\overset{\leftarrow }{h}}_{t}$ are fused to yield the final hidden state $h_{t}$, which incorporates bidirectional information as follows:

(5) $$h_{t}=[{\vec{h}}_{t},{\overset{\leftarrow }{h}}_{t}]$$

BiGRU combines the processing capability of long-range dependencies with bidirectional information capture. It inherits the gating mechanism of the GRU to prevent gradient vanishing, enabling the effective capture of distant dependencies within long sequences. Compared with unidirectional GRU and LSTM, it simultaneously utilises past and future contexts to achieve bidirectional information fusion. It has fewer parameters than BiLSTM and structurally simplifies LSTM by removing the cell state and output gate, thereby reducing the computational complexity.

## 3. Methodologies

### 3.1. Model Construction

**Figure 1:**
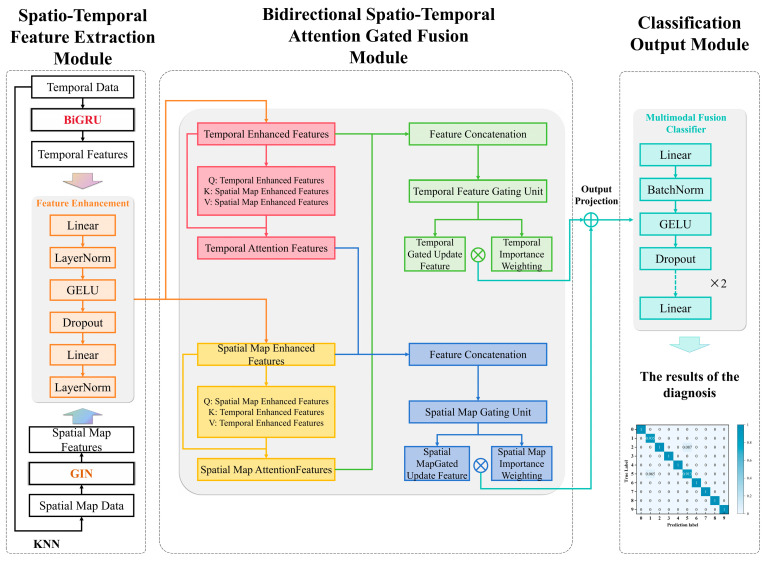
The architecture of spatio-temporal feature fusion model.

The spatio-temporal feature fusion model STFFM comprises three components: a spatio-temporal feature extraction module, a bidirectional spatio-temporal attention-gated fusion module, and a classification output module. The model architecture is illustrated in Figure 1.

(1) Spatio-Temporal Feature Extraction Module

The spatio-temporal feature extraction module comprises two components: temporal feature extraction and spatial graph feature extraction. Feature enhancement components augment the expressive power of spatio-temporal features, thereby improving their discriminative capability.

For temporal feature extraction, this study employs BiGRU to capture temporal dependencies within long sequences. Unlike conventional BiGRU implementations, which typically stack one or two layers without batch normalisation, the structure has been enhanced through two key innovations: on the one hand, a multi-layered bidirectional GRU architecture with two units per layer, enhancing the hierarchical modelling of temporal patterns; on the other hand, BatchNorm1d is introduced after each GRU layer, with dimension transposition mitigating instability during deep network training. The input data were first reshaped into fixed-dimensional tensors, while outputs were selected from the feature representations at the final time step. Dropout is then applied to these outputs, thereby enhancing the local feature extraction capabilities and the expression robustness of the model. The BiGRU network architecture employed in this study is described in Table 1.

In the spatial graph feature extraction section, this study introduces a graph structure construction method based on KNN, which converts one-dimensional time-series signals into graph structure data. Subsequently, a GIN is employed to effectively capture spatial domain dependencies. Specifically, for each bearing vibration signal sample, each sampling point was treated as a node in the graph, with the node feature being the signal value at that point. Subsequently, the KNN algorithm calculates the five nearest neighbours for each node within the feature space, establishing edge connections accordingly. This ultimately transforms each sample into a graph data object comprising node features, edge structure, and corresponding fault type labels, thereby constructing a graph dataset suitable for training graph neural networks. Building on this, the GIN network performs hierarchical feature extraction on graph data, progressively aggregating node information through multi-layer graph convolutional operations to generate graph-level feature representations. Compared to the conventional MLP architecture in traditional GIN, which predominantly employs stacked linear layers, this study introduces enhancements: one dimensional batch normalisation layer (BatchNorm1d), Gaussian Error Linear Units (GELU) activation functions, and dropout mechanisms are incorporated into the MLP corresponding to each GINConv layer. Batch normalisation is applied after each graph convolutional layer to enhance feature distribution stability and improve the model’s resistance to overfitting. Concurrently, the network adopts a dimension design that progressively increases from 1→128→256→512 across layers. Combined with multi-layer feature extraction and dropout operations following global mean pooling, this further strengthens the hierarchical nature and robustness of the graph feature representations. The GIN network architecture employed in this study is illustrated in Table 2.

(2) Bidirectional Spatio-Temporal Attention Gate Fusion Module

To achieve bidirectional weighted fusion of spatio-temporal features, a bidirectional multi-head attention mechanism and a cross-modal gate update mechanism are employed for dynamic feature updating and adaptive fusion.

Within the bidirectional multi-head attention mechanism, multimodal attention features are derived from Equations (6) and (7):

(6) $${\mathrm{Seq}}_{\mathrm{attn}}=\mathrm{MHA}(Q=\mathrm{Graph},K=V=\mathrm{Seq})$$

(7) $${\mathrm{Graph}}_{\mathrm{attn}}=\mathrm{MHA}(Q=\mathrm{Seq},K=V=\mathrm{Graph})$$

where MHA denotes the multi-head attention mechanism function, *Q* represents the query vector, *K* denotes the key vector, *V* signifies the value vector, Seq denotes the sequence-augmented feature vector, and Graph denotes the spatial graph-augmented feature vector. Temporal attention features ${\mathrm{Seq}}_{\mathrm{attn}}$ use graph features as the query, employing temporal features as key-value pairs for attention computation, thereby yielding graph-enhanced temporal features. Conversely, spatial graph attention features ${\mathrm{Graph}}_{\mathrm{attn}}$ employ temporal features as queries, utilising graph features as key-value pairs for attention computation, thus generating graph-enhanced temporal features.

The weights of the gating units in the cross-modal gated update mechanism are obtained from Equations (8) and (9):

(8) $$G_{\mathrm{seq}}=\sigma (\mathrm{LayerNorm}(W_{\mathrm{seq}}[{\mathrm{Seq}}_{\mathrm{enhanced}}\|{\mathrm{Graph}}_{\mathrm{attn}}]))$$

(9) $$G_{\mathrm{graph}}=\sigma (\mathrm{LayerNorm}(W_{\mathrm{graph}}[{\mathrm{Graph}}_{\mathrm{enhanced}}\|{\mathrm{Seq}}_{\mathrm{attn}}]))$$

where σ denotes the Sigmoid activation function, LayerNorm represents layer normalisation operations, $W_{\mathrm{seq}}$ and $W_{\mathrm{graph}}$ denote linear transformation weight matrices, [·||·] denotes vector concatenation operations, and ⊙ denotes element-wise multiplication. The temporal gating weight *G*_seq_ serves as the gating vector that controls the proportion of temporal features retained, whereas the spatial graph gating weight *G*_graph_ acts as the gating vector that controls the proportion of graph features retained. Higher values indicate greater preservation of the original features.

Upon obtaining the gating weights, the multimodal features undergo gated updates according to Equations (10) and (11):

(10) $${\mathrm{Seq}}_{\mathrm{updated}}=G_{\mathrm{seq}}⊙{\mathrm{Seq}}_{\mathrm{enhanced}}+(1-G_{\mathrm{seq}})⊙{\mathrm{Graph}}_{\mathrm{attn}}$$

(11) $${\mathrm{Graph}}_{\mathrm{updated}}=G_{\mathrm{graph}}⊙{\mathrm{Graph}}_{\mathrm{enhanced}}+(1-G_{\mathrm{graph}})⊙{\mathrm{Seq}}_{\mathrm{attn}}$$

Here, the Seq_updated_ represents a weighted combination of temporal augmentation and spatial graph attention features, whereas the Graph_updated_ constitutes a weighted integration of graph augmentation and temporal attention features.

To achieve adaptive fusion of the Seq_updated_ and Graph_updated_ features, learnable temporal importance weight parameters *α*_seq_ and spatial graph importance weight parameters *α*_graph_ are introduced. These parameters were learned during model training, ultimately enabling weighted fusion according to Equation (12). The spatio-temporal fusion feature *F*_fused_ is then output via a projection layer, as expressed in Equation (13):

(12) $$\mathrm{Output}=\alpha_{\mathrm{seq}}\cdot {\mathrm{Seq}}_{\mathrm{updated}}+\alpha_{\mathrm{graph}}\cdot {\mathrm{Graph}}_{\mathrm{updated}}$$

(13) $$F_{\mathrm{fused}}=\mathrm{Linear}(\mathrm{Dp}(\mathrm{GELU}(\mathrm{LN}(\mathrm{Linear}(\mathrm{Output}))))$$

Here, Linear denotes a fully connected layer, Dp represents a dropout layer, GELU signifies the activation function, and LN indicates a LayerNorm layer.

(3) Classification Output Module

The multimodal feature fusion classifier comprises two identically structured layers that are connected in series. Each structure incorporates a fully connected layer (linear), a BatchNorm1d, a GELU activation function, and a dropout layer. Through dimension reduction and multilayer regularisation, this design prevents the loss of high-dimensional feature information while enhancing the model’s generalisation capability for fault classification tasks. Finally, a fully connected layer is used to implement the classification output of the multimodal fused features.

### 3.2. Steps of Fault Diagnosis

The main steps of the spatio-temporal feature fusion model constructed in this paper for reciprocating compressor fault diagnosis are as follows:

(1) Fault data acquisition and preprocessing: Multi-sensor time-series signals are collected from reciprocating compressors, and the sliding window method is employed to augment the sample size.
(2) Constructing Graph Structures via KNN Algorithm: Each time-series sample is treated as a graph node. The Euclidean distance or cosine similarity is calculated between samples, with K nearest neighbours per node forming edges weighted by similarity values. The original fault labels were retained as node labels.
(3) Dataset partitioning: Hierarchical sampling is employed to proportionally divide the merged temporal and graph samples into training, validation, and test sets. This ensured a consistent fault category distribution across all sets, mitigating the model evaluation bias caused by class imbalance.
(4) Model Construction and Training: A spatio-temporal feature fusion model is constructed. BiGRU is employed to extract temporal features, whereas GIN is used to extract spatial features for feature fusion. Cross-modal feature fusion is achieved using a spatio-temporal bidirectional attention gating module. The output is mapped to the fault category space via a fully connected layer to produce the classification results.
  During training, the training set was inputted, employing the cross-entropy loss function and AdamW optimiser for parameter updates. The model performance is evaluated on the validation set after each iteration, monitoring changes in loss and accuracy to save the model weights, yielding optimal validation set performance.
(5) Model Testing and Evaluation: The optimal model is loaded to perform fault diagnosis inference on the test set. The model performance was assessed using metrics such as classification accuracy and precision. Combined with TSNE visualisation analysis of the class separability of the fused features, this validates the model’s capability to identify compressor faults.

**Figure 2:**
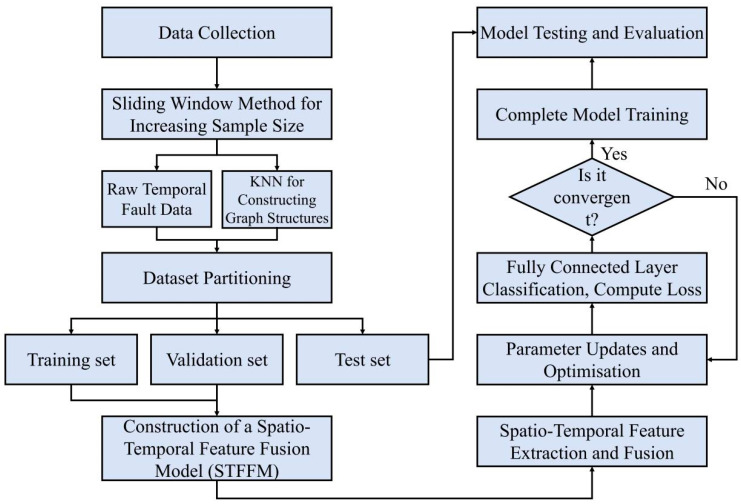
Fault diagnosis flowchart.

The fault diagnosis flowchart is shown in Figure 2.

## 4. Case Study

### 4.1. Data Sources

Fault diagnosis for reciprocating compressors aims to address the challenges of low utilisation rates for sensor-collected data and the difficulty in extracting fault characteristics within industrial settings. Its core premise relies on signal acquisition from multiple sensor types, including vibration and temperature sensors, to capture time-series data during equipment operation.

**Figure 3:**
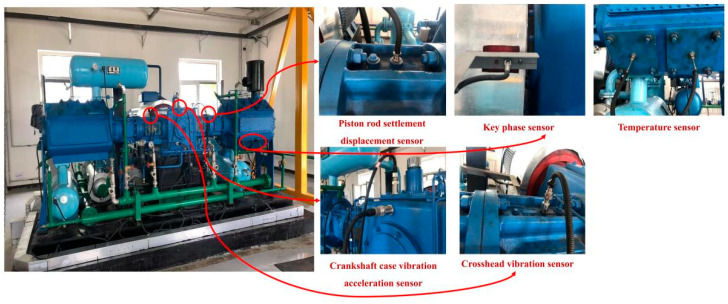
Data Acquisition for reciprocating air compressors.

Experimental data were collected from a DWF-10/6 twin-cylinder reciprocating air compressor, as illustrated in Figure 3. The main motor power was 75 kW, with fault data acquired online via sensors. The fault dataset comprises two sets of data: vibration settlement and temperature-speed. The vibration settlement data include six channel measurements: crankcase vibration, cylinder housing vibration, cylinder settlement, etc. The temperature-speed data encompassed four channels, including the cylinder discharge temperature.

The data acquisition system was configured with a sampling frequency of 10,240 Hz, 6144 sampling points for piston rod settlement and casing vibration, and 5140 sampling points for crankshaft case vibration.

As typical faults in reciprocating compressors, such as valve impact and component wear friction, often contain rich high-frequency components, the frequency domain distribution characteristics shown in Section 4.2 corroborate this observation. Key fault types under investigation, such as valve faults, exhibit characteristic frequencies reaching 5 kHz. According to the Nyquist sampling theorem, the sampling frequency must be at least twice the target characteristic frequency. To avoid frequency aliasing, ensure the capture of high-order harmonic information from fault characteristics, and reserve a sufficient margin for subsequent frequency domain analysis, 10,240 Hz was ultimately selected as the system sampling frequency.

Furthermore, the reciprocating compressor employed in this experiment operated at a rated speed of 600 rpm, corresponding to a single rotational cycle of 0.1 s. Equipment fault characteristics typically manifest as patterns that are integer multiples of these cycles. The number of sampling points was set to 6144 for both the piston rod deflection and casing vibration signals. This parameter represents the default hardware configuration of the experimental data acquisition system for the piston-rod deflection and casing vibration measurement channels. Combined with the sampling frequency, this yielded a sampling duration of 0.6 s, covering six complete compressor rotation cycles. This duration sufficiently captures the time-domain evolution characteristics of periodic faults, such as piston ring wear and valve impact, effectively ensuring the resolution for the combined time- and frequency-domain analyses. The sampling point count for the crankshaft case vibration signal was set to 5140. This parameter is constrained by the storage capacity limit of the hardware buffer on the data acquisition card for the channel on the test bench. Calculations indicated a sampling duration of approximately 0.5 s, covering five complete rotation cycles. Considering that faults such as crankshaft bearing wear and connecting rod bolt loosening exhibit higher stability in their characteristic cycles, the sampling data from five complete cycles sufficiently supported subsequent fault feature extraction and pattern recognition without compromising the validity of the analytical results. Although the sampling point counts differ between the two parameters, both satisfy the redundancy design requirement of covering multiple complete operating cycles, thereby ensuring the scientific rigor and reliability of fault feature extraction.

**Figure 4:**
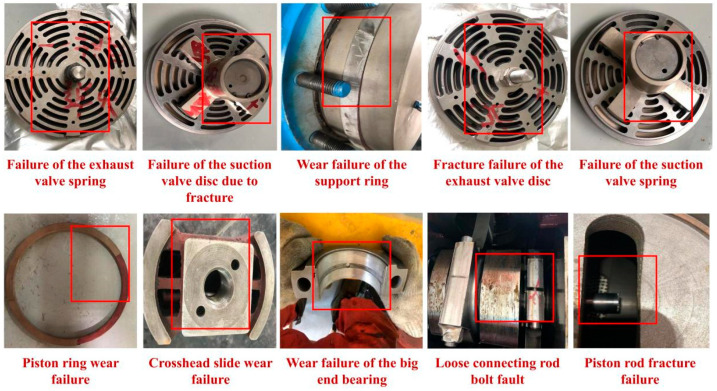
Fault mode characterisation.

Five single-fault mode datasets, four composite-fault mode datasets, and one set of normal operating data were collected. The fault mode characterisation is illustrated in Figure 4. All fault data were acquired under identical operating conditions.

The aforementioned instructions for sensor parameter configuration may serve as a reference for sensor data acquisition in relevant mechanical equipment.

The study utilised vibration and settlement data from the fault dataset to construct a 10-class classification dataset encompassing normal data, five single-fault modes, and four composite fault modes. The analysis was conducted using cylinder shell vibration channel signals, with the dataset partitioning and labelling detailed in Table 3.

### 4.2. Data Preprocessing

**Figure 5:**
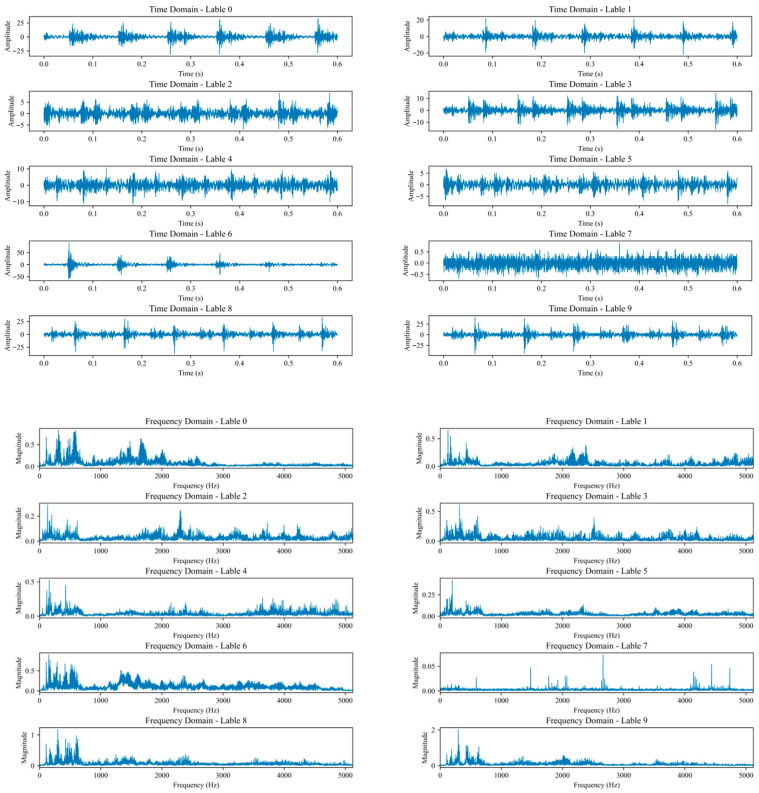
Time-domain and frequency-domain signal plots for Labels 0–9.

Owing to the substantial noise generated during reciprocating compressor operation, obtaining a high-performance pre-trained model necessitates sufficient fault samples for training. The experiment employed a sliding window approach for data processing, with each sample comprising 1024 sampling points. The time step was set to 1024, and the sampling window overlap rate was 50%. A total of 2330 samples were acquired across the 10 fault modes. These were partitioned in a 7:2:1 ratio to yield 1631 training samples, 466 validation samples, and 233 test samples. Figure 5 shows the time-domain signal plots for all the fault modes.

### 4.3. Analysis of Experimental Results

To validate the fault diagnosis capability of the proposed method, a systematic analysis of the performance of the model on the test set was conducted using standard classification evaluation metrics. The experimental test dataset comprised 233 samples categorised into 10 classes, with each class containing a balanced distribution of 19–31 samples. This study employed the average of the final 10 epochs from each training run as the result of a single round of experiments, thereby mitigating experimental errors caused by variability within a single round. Five single-round experiments were conducted, and the average of these five results was taken as the final experimental outcome to reduce experimental errors stemming from variability across multiple rounds. The diagnostic results are presented in Table 4.

Table 4 demonstrates that the proposed method exhibited outstanding performance in the 10-classification task, achieving an overall accuracy of 99.14% across 233 test samples, with a macro-average F1-score of 0.9917 and a weighted average F1-score of 0.9915. This validates the balanced adaptation of the model across all fault modes. Furthermore, the model demonstrates outstanding performance for the four composite fault modes labelled 6–9, indicating that the proposed method effectively integrates spatio-temporal feature information to address fault diagnosis challenges in reciprocating compressors under composite fault scenarios.

**Figure 6:**
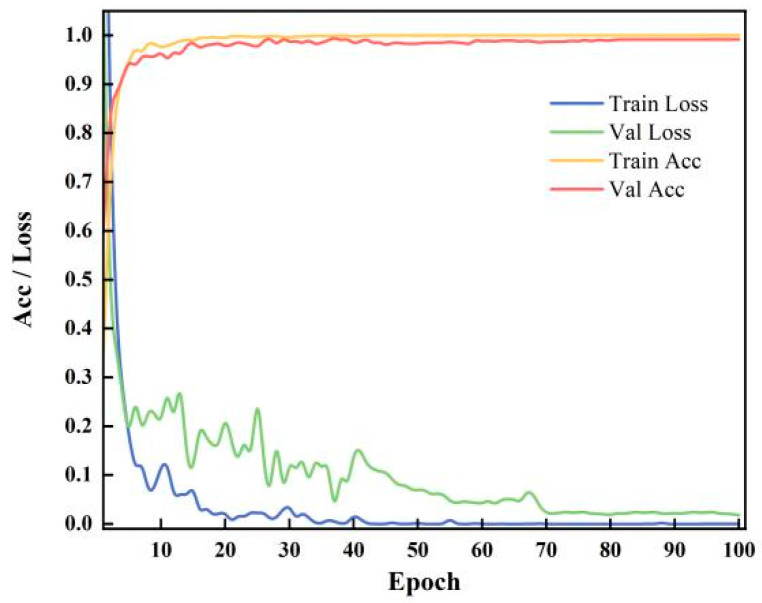
Performance evolution curve.

To investigate the model’s performance evolution during training and validation, accuracy versus loss curves were plotted, as shown in Figure 6. Both the training and validation losses exhibited a monotonically decreasing trend with increasing epochs. Between 0 and 30 epochs, the model’s accuracy significantly improved, while loss rates substantially decreased, indicating a rapid learning phase. Around 30 epochs, the model entered a convergence phase, engaging in deep learning of spatio-temporal feature details. After 100 epochs of training, the accuracy and loss curves converged consistently with no evident signs of overfitting.

**Figure 7:**
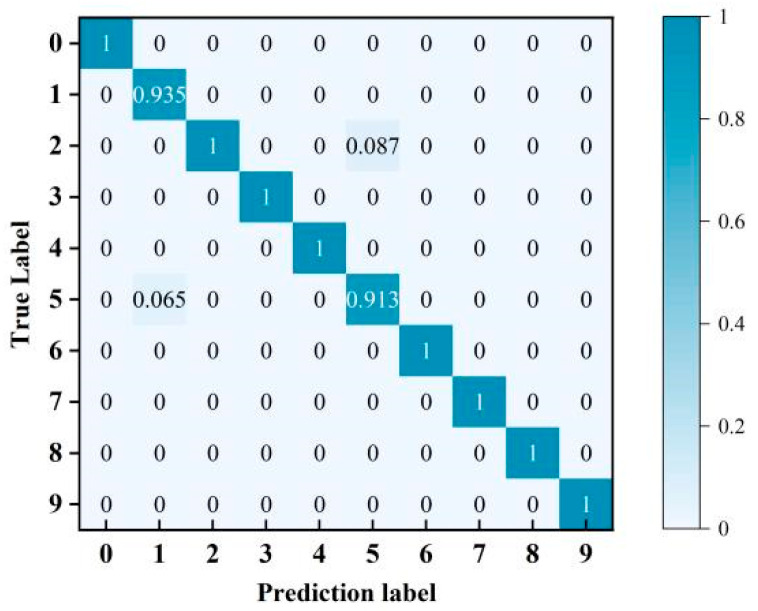
Confusion matrix.

To further investigate the performance of the proposed method on the test set, a confusion matrix was introduced to evaluate the model. The vertical axis represents the true labels, the horizontal axis represents the predicted labels, and the diagonal data indicate classification accuracy. The diagnostic confusion matrix normalised by the true labels is shown in Figure 7.

As shown in Figure 7, the correct classification (diagonal elements) of prediction results across various fault models accounts for a high proportion, whereas misclassification (non-diagonal elements) constitutes a low proportion. The proposed method achieves a diagnostic accuracy exceeding 90% for all fault modes, demonstrating its capability for precise fault-type classification.

**Figure 8:**
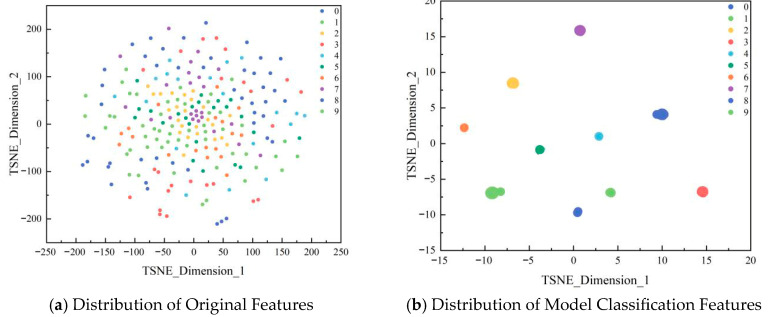
T-SNE visualisation: (**a**,**b**).

Finally, to further elucidate the feature representation process, Figure 8 shows t-distributed stochastic neighbourhood embedding (t-SNE) [17] for feature visualisation, with different colours representing distinct fault mode characteristics. Figure 8a depicts the original feature distribution of the dataset, whereas Figure 8b shows the feature distribution after model classification.

Figure 8a demonstrates that the raw signal features exhibit severe aliasing in two-dimensional space, with samples from different fault categories becoming entangled. The feature distributions of compound and single faults overlap significantly, making effective differentiation through simple linear or nonlinear decision boundaries challenging. This indicates that reciprocating compressor fault signals exhibit strong coupling characteristics. Relying solely on raw time-domain features fails to meet the demands of high-precision fault diagnosis, highlighting the necessity of deep feature extraction.

In contrast to Figure 8b, after processing using the STFFM model, features from different fault modes exhibit pronounced clustering effects. The intra-class compactness and inter-class separability were significantly improved. The comparison of the two feature distributions validates the effectiveness of the proposed model at the feature learning level. By employing BiGRU to uncover temporal dependencies within signals, utilising GIN to capture spatial domain feature differences, and integrating a bidirectional attention gating mechanism to achieve precise cross-modal feature fusion, the proposed approach effectively separates redundant information and noise interference from raw signals. This enables the extraction of highly discriminative spatio-temporal coupled features, addressing the shortcomings of traditional methods in spatio-temporal feature extraction and the neglect of spatio-temporal correlations by existing models.

## 5. Discussion

### 5.1. Model Ablation

**Figure 9:**
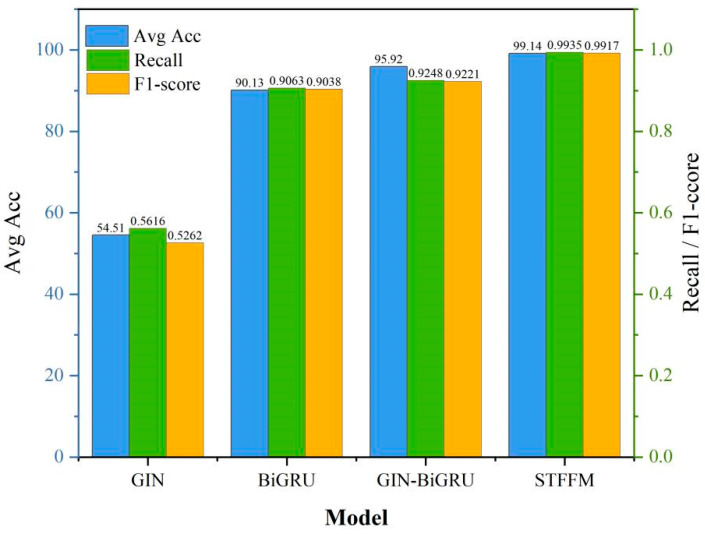
Model ablation experiment results.

To further investigate the mechanisms and effectiveness of each module within the proposed methodology, model ablation experiments were conducted. All experimental settings remained consistent with those described earlier in this paper; the experimental setup is shown in Table 5, and the ablation results are presented in Figure 9.

Figure 9 clearly demonstrates the contribution of each module of the proposed method to the model’s performance. The experimental results revealed that the M1 model, which utilised only the GIN module, exhibited the lowest performance, with an accuracy of only 54.51%. This indicates that pure graph structural information has significant limitations when addressing sequential signal classification tasks. Conversely, the M2 model, which employs only the BiGRU module, achieves an accuracy of 90.13%, thereby validating the pivotal role of temporal modelling in vibration signal classification. The M3 model, which combines GIN and BiGRU, further elevated the accuracy to 95.92%, demonstrating that graph structural information provides valuable supplementary features for temporal models and fully proves the complementary nature of spatio-temporal features. The M4 model, which incorporates the spatio-temporal feature fusion module (STFFM), achieved an accuracy of 99.14%, significantly outperforming other ablation models across all evaluation metrics. This validates the crucial role of the STFFM module in spatio-temporal feature fusion and demonstrates the effectiveness of the proposed GIN-BiGRU-STFFM architecture in capturing complex spatio-temporal patterns. Compared with other ablation models, the proposed method achieves accuracy improvements of 44.63%, 7.01%, and 3.22%, respectively. Ablation experiments demonstrated that each model component substantially contributed to the final performance improvement, with significant synergistic effects observed between the modules.

### 5.2. Comparison of Different Methods

To further validate the advantages of the proposed method over existing approaches, an analysis and comparison of different methodologies was conducted. Table 6 presents comparative results.

The comparative experimental results conclusively demonstrate the significant advantages of the proposed method for diagnosing reciprocating compressor faults. It outperforms traditional approaches such as SSA-KELM, Improved MEEMD-SqueezeNet, and AVSMF by 10.61, 4.97, and 2.07 percentage points, respectively, while surpassing advanced methods such as MPMRNet and T-DBN by 0.86 and 7.14 percentage points, respectively. This highlights its substantial superiority in terms of diagnostic accuracy. The findings reflect the efficacy of spatio-temporal feature fusion mechanisms in processing complex vibration signals. Compared to approaches such as T-DBN and SSA-KELM, which focus solely on temporal features, and methods such as MPMRNet, which possess strong feature extraction capabilities but fail to fully exploit spatio-temporal correlations, the proposed STFFM model achieves deep excavation and effective integration of latent spatio-temporal patterns within vibration signals. This is accomplished through the organic combination of graph inference networks with bidirectional temporal modelling, alongside the introduction of a spatio-temporal feature fusion module. Consequently, the model demonstrated outstanding performance in fault diagnosis tasks.

## 6. Conclusions

This paper addresses the inadequacies of traditional spatio-temporal feature extraction methods in reciprocating compressors and the neglect of spatio-temporal correlations in existing modelling approaches. A spatio-temporal feature fusion model was constructed and validated using an industrial dataset from reciprocating compressors, demonstrating its effectiveness and feasibility. The following conclusions were drawn:

(1) Through the synergistic operation of the spatio-temporal feature extraction module, spatio-temporal dual-selection attention-gated fusion module, and classification output module, the model effectively extracts and integrates spatio-temporal correlation information of faults, achieving precise fault diagnosis, with an average accuracy rate of 99.14%.
(2) Model ablation experiments validated the complementary nature of spatio-temporal information. The enhanced BiGRU design effectively strengthens the extraction of temporal dependencies in long-term sequence data, while the modified graph isomorphism network (GIN) effectively captures spatial variations in fault information. Integrating these through spatio-temporal bidirectional attention gating achieves a dynamic complementary fusion of spatio-temporal features. The resulting GIN-BiGRU-STFFM model demonstrated accuracy improvements of 44.63%, 7.01%, and 3.22% over GIN, BiGRU, and GIN-BiGRU, respectively.
(3) The spatio-temporal feature fusion model effectively captures temporal and spatial graph domain information, demonstrating significant superiority over other network architectures. It achieves performance gains of 10.61%, 4.97%, and 2.07% over the traditional SSA-KELM, Improved MEEMD-SqueezeNet, and AVSMF methods, respectively, while outperforming the state-of-the-art MPMRNet and T-DBN approaches by 0.86% and 7.14%, respectively.

## Figures and Tables

**Table 1:** BiGRU network architecture employed in this paper.

Layer	Component	Input Dimensions	Output Dimensions
Input Layer	Sequential Signal Preprocessing	32	(32, 32)
Layer 1 BiGRU	BidirectionalGRU + BatchNorm + GELU	(32, 32)	(32, 256)
Layer 2 BiGRU	BidirectionalGRU + BatchNorm + GELU	(32, 256)	(32, 512)
Output Layer	Final Time Step + Dropout + Dropout	(32, 512)	512

**Table 2:** GIN network architecture employed in this paper.

Layer	Component	Input Dimensions	Output Dimensions
Input Layer	Primitive Node Characteristics	1	1
Layer 1 GINConv	MLP + GINConv + BatchNorm + GELU	1	128
Layer 2 GINConv	MLP + GINConv + BatchNorm + GELU	128	256
Layer 3 GINConv	MLP + GINConv + BatchNorm + GELU	256	512
Output Layer	Global Average Pooling + Dropout	512	512

**Table 3:** Dataset Partitioning and Labelling.

Fault Type	Label	Fault Name
Single fault mode	1	Cylinder 1 failure of the exhaust valve spring
	2	Cylinder 1 failure of the suction valve disc due to fracture
	3	Cylinder 1 wear failure of the support ring
	4	Cylinder 2 fracture failure of the exhaust valve disc
	5	Cylinder 2 failure of the suction valve spring
Compound failure mode	6	Cylinder 1 piston rod fracture failure & Cylinder 2 piston ring wear failure
	7	Cylinder 1 crosshead slide wear failure & Cylinder 2 piston ring wear failure
	8	Cylinder 2 wear failure of the big end bearing & Cylinder 1 crosshead slide wear failure
	9	Cylinder 2 loose connecting rod bolt fault & Cylinder 1 crosshead slide wear failure
Normal mode	0	Normal

**Table 4:** Diagnosis results.

Label	Precision	Recall	F1-Score	Support
0	1.0000	1.0000	1.0000	26
1	1.0000	0.9355	0.9667	31
2	1.0000	1.0000	1.0000	24
3	1.0000	1.0000	1.0000	24
4	0.9048	1.0000	0.9500	19
5	1.0000	1.0000	1.0000	23
6	1.0000	1.0000	1.0000	19
7	1.0000	1.0000	1.0000	24
8	1.0000	1.0000	1.0000	21
9	1.0000	1.0000	1.0000	22
Accuracy	-	-	0.9914	233
Macro avg	0.9905	0.9935	0.9917	233
Weighted avg	0.9922	0.9914	0.9915	233

**Table 5:** Model ablation experiment results.

Model Number	Ablation Model Name	Parameter Settings
M1	GIN	Learning rate: 0.001 batch size = 32 optimiser: AdamW Epoch = 100
M2	BiGRU	
M3	GIN-BiGRU	
M4	STFFM	

**Table 6:** Comparison of different methods.

Method	Average Accuracy	Reference	Method Introduction
SSA-KELM	88.53%	Tang et al. 2023 [9]	Multi-source signal fusion
Improved MEEMD-SqueezeNet	94.27%	Zhang et al. 2023 [18]	Combining multi-scale information fusion with deep learning
MPMRNet	98.28%	Zhang et al. 2021 [19]	Residual network diagnostic method
T-DBN	92.00%	Jiang et al. 2025 [20]	Feature transfer method
AVSMF	97.07%	Fang et al. 2024 [21]	Adaptive variable-scale shape filter
STFFM	99.14%	Proposed Method	Spatio-temporal feature fusion

## Data Availability

The datasets generated and analysed during the current study are available from the authors upon reasonable request.
